# Measuring Social and Emotional Wellbeing in Aboriginal Youth Using Strong Souls: A Rasch Measurement Approach

**DOI:** 10.3390/ijerph18168425

**Published:** 2021-08-10

**Authors:** Ella Gorman, Brody Heritage, Carrington C. J. Shepherd, Rhonda Marriott

**Affiliations:** 1 Discipline of Psychology, Murdoch University, Perth, WA 6150, Australia; Brody.Heritage@telethonkids.org.au; 2 Ngangk Yira Research Centre for Aboriginal Health and Social Equity, Murdoch University, Perth, WA 6150, Australia; carrington.shepherd@curtin.edu.au (C.C.J.S.); R.Marriott@murdoch.edu.au (R.M.); 3 Telethon Kids Institute, The University of Western Australia, Perth, WA 6009, Australia; 4 Curtin Medical School, Curtin University, Perth, WA 6102, Australia

**Keywords:** Rasch, Aboriginal, youth, social and emotional wellbeing, psychometrics

## Abstract

Currently, there are few robustly evaluated social and emotional wellbeing (SEWB) measures available for use with Aboriginal youth in research, policy, and practice. As such, this study used a Rasch measurement approach to examine the psychometric properties of Strong Souls, a 25-item self-reported SEWB instrument, created for use with Aboriginal youth in the Northern Territory. Our sample (*N* = 154) included youth (15–25 years old) living on Whadjuk (metropolitan Western Australia; *N* = 91) and Kamilaroi countries (rural New South Wales; *N* = 63). Using Rasch modelling techniques, evidence for multidimensionality in the scale was observed, resulting in subsequent analyses conducted separately on two subscales: Psychological Distress and Resilience. The Resilience subscale did not meet the Rasch model assumptions, with poor person and item separation and reliability indexes suggesting the scale was not reliably differentiating between participants’ Resilience scores. The Psychological Distress subscale had mixed separation and reliability index results, with good construct validity implied but poorer ability to target the distress of participants. Our findings provide novel evidence demonstrating the functioning of Strong Souls in a contemporary sample of Aboriginal youth, suggesting further modifications of the instrument are required before it can be used with confidence as a reliable measure in this population group.

## 1. Introduction

Adolescence and young adulthood are key transition periods in the lifespan that present opportunities and challenges. This period lays the foundation for future employment, health and relationship outcomes into adulthood and later life [1]. In recent decades, rapid societal changes involving technology, the environment, and economic fluctuations have added further complexities for young people navigating their way to adulthood. Aboriginal and Torres Strait Islander (herein respectfully referred to as Aboriginal) youth face unique complexities and challenges compared with other young people in successfully transitioning to adulthood [2]. It is well-established that many disparities exist between Aboriginal and non-Aboriginal people in Australia. These disparities are linked to destructive colonial mechanisms involving many years of cultural erasure and land dispossession, forced removal of children from their families, and the resulting, complex intergenerational trauma [3]. Institutionalised attitudes, policies, and structures remain in place that maintain and mirror this past, and continue to impact Aboriginal people today [4]. Given the particularly young age structure of Aboriginal peoples in Australia (the median age being 23 years compared to 37.8 years for non-Indigenous Australians [5]), the impact that this socio-political context has on Aboriginal youth and adolescents is particularly pressing. Policies and programs that are responsive to not only this context but also the needs of young Aboriginal people specifically, are necessary to support and the strengthen and wellbeing of this population [2].

Much of the existing policy and reporting frameworks regarding Aboriginal health and wellbeing continue to be deficit-focussed. While a core national focus on the *Closing the Gap* campaign has reported some improvements in areas such as education and infant mortality [6], the framework remains centred around comparison between Aboriginal and Torres Strait Islander peoples and the non-Indigenous population, often furthering the deficit-focussed narrative [7]. The focus on ‘the gap’ often provides limited information as to where and how to improve Aboriginal health and wellbeing, and can mask real improvement and progress [8,9]. As such, the prevailing discourse regarding Aboriginal health does not tend to feature the breadth of strengths that underpin Aboriginal cultures, nor frame the experiences of Aboriginal peoples as one of survival and resilience, even in the context of pervasive disadvantage [10,11]. This also applies to the domain of mental health, where a holistic notion of wellbeing (social and emotional wellbeing; SEWB) is central to an Aboriginal worldview.

Gee et al. [12] defined SEWB as “…a multidimensional concept of health that includes mental health, but also encompasses domains such as connection to land or Country (“Country” has been used as a proper noun by deliberate choice—the term “connection to Country” in this context refers to Aboriginal and Torres Strait Islander peoples’ relationship to traditional ancestral lands. This extends beyond a Western notion of country as the geographic location specifically, to also include a multilayered and reciprocal spiritual connection. Please see Chapter 1 - Aboriginal Social, Cultural and Historical Contexts (pp. 3–24 from *Working Together: Aboriginal and Torres Strait Islander Mental Health and Wellbeing Principles and Practices* [13] for a comprehensive overview), culture, spirituality, ancestry, family and community” (p. 55). This collectivist view holds the individual as embedded and inseparable from these interconnected and overlapping domains of SEWB, intertwined with social and historical determinants of health. Thus, for health-related research to be relevant and valuable for Aboriginal people, it is essential for researchers and relevant professionals to use this understanding of SEWB to guide practice and policy. As noted by Zubrick et al. [14], Aboriginal health policy has previously been rooted in a ‘mental health’ framework, only using the measurement of severe mental health outcomes as indicators of wellbeing. More recent policy approaches such as the *National Strategic Framework for Aboriginal and Torres Strait Islander Peoples’ Mental Health and Social and Emotional Wellbeing* [15] demonstrate a slow shift in some areas towards a more strengths-based approach to wellbeing.

### 1.1. Measuring SEWB in Aboriginal People

Having the means to accurately measure a concept such as SEWB is critical to be able to gather accurate information to inform and evaluate the effectiveness and outcomes of policies and programmes designed to support young peoples’ SEWB. There is a paucity of quality measures available to assess SEWB among Aboriginal youth [16] despite the fact that they: (a) experience different and disproportionate risks for poorer mental wellbeing outcomes; and (b) may not identify with Western perspectives of mental wellbeing. Mainstream instruments, which may frame interpretation of their results in a deficit-based perspective, have previously been employed in research and practice without validation for Aboriginal populations (e.g., Assessment of Quality of Life-4D; [17]). Other measurement methods have been modified or adapted to an Aboriginal context, such as the Patient Health Questionnaire 9 [18]. Le Grande et al. [19] have recently reviewed the quality of instruments used to measure SEWB in Aboriginal samples, and found that very few SEWB instruments have been developed specifically for and with Aboriginal people. Even less research has focussed on the use and appropriateness of SEWB measures in Aboriginal youth, rather than adult populations (see Thurber et al., [20]). One of the few instruments that has been developed explicitly for use with Aboriginal youth to measure SEWB is Strong Souls [21].

### 1.2. Strong Souls

Strong Souls was developed in response to a lack of appropriate tools available to assess the SEWB of Aboriginal youth in a longitudinal birth cohort study in the Northern Territory (NT; Aboriginal Birth Cohort [ABC] Study; [21]). The development of the 25 items that comprise the final scale were initially informed by a literature review into “Indigenous and mental health literature” with a focus on resilience, depression, anxiety and suicide risk as relevant factors, along with input from an Aboriginal consultancy group comprised of community members and mental health professionals. An initial pilot test with a small sample of Aboriginal youth (*n* = 67) compared the discriminative power and reliability of an initial 34 items with that of two relevant, established scales: Kessler Psychological Distress (K6+; [22]) and the Westerman Aboriginal Symptoms Checklist for Youth (WASC-Y; [23]). This approach resulted in the final 25 items, split across four latent factors or subscales: depression (7 items), anxiety (6 items), suicide risk (3 items) and resilience (9 items). Using exploratory factor analysis, the initial pilot study (*n* = 361) reported good construct validity, reliability, acceptability and cultural appropriateness for use with its sample of youth in the NT (aged between 16–20.5 years; [21]). However, no further studies evaluating the full scale’s psychometric properties have been published since Thomas et al. [21]. This absence of information limits the ability of researchers and practitioners to use the instrument with confidence in other samples and sub-populations.

This lack of further study on Strong Souls is significant, particularly given the population heterogeneity across and between different jurisdictions in a country as large as Australia. Importantly, this is demonstrated in the cultural and historical diversity between the many hundreds of distinct Aboriginal and Torres Strait Islander Nations (e.g., Noongar, Kamilaroi) that exist across the continent [24]. Furthermore, the NT is unique within the Australian states and territories in that around 30% of the population identifies as Aboriginal and/or Torres Strait Islander. In comparison, New South Wales (NSW) and Western Australia (WA) have an Aboriginal population rate of around three percent—closer to the national Indigenous population proportion [5]. Additionally, at 77%, a far greater proportion of the Aboriginal NT population live in remote or very remote areas compared to other states and territories; WA, for example, has the second highest proportion of Aboriginal people living in remote or very remote areas, at 38% [5]. As such, generalising the findings from the original examination of Strong Souls [21], and using the scale with different cohorts from contrasting parts of the country, would not necessarily be appropriate nor produce reliable or valid results.

### 1.3. Measurement—Psychometrics

In consideration of the validity of scores derived from quantitative measurement tools, there are several methods available to evaluate their validity. Classical test theory (CTT) or true score theory approaches are most common in the literature regarding measurement validation. However, there is increasing recognition that modern approaches to scale development and evaluation, such as Rasch analysis (a restricted form of Item Response Theory modelling), are advantageous in addressing knowledge gaps that CTT is unable to cover when examining the properties of a measure’s scores [25].

Rasch analysis is a modelling approach where participant scale responses are used to locate participants along a latent continuum from low to high on the scale construct (in this case, SEWB). Indeed, one of the strengths of the Rasch measurement approach is the capacity to isolate the person abilities (i.e., participants’ level of SEWB) separate to that of estimates of the difficulties of the items themselves [26]. The latter affords scrutiny regarding the range of SEWB being measured by the instrument and by which items (i.e., is there a ceiling effect and the measure lacks sensitivity to high levels of SEWB based on participant scores?), informing practitioners wishing to use the measure as a screening tool for SEWB (i.e., can the scores accurately identify persons who may be in the lower range for SEWB?). This differs from CTT, where item difficulty estimates are dependent on the sample [25].

Furthermore, a primary assumption of the Rasch model is that scores should reflect a unidimensional construct—that is, the instrument is only measuring one thing. This assumption is particularly pertinent in the context of the Strong Souls, given its advised scoring method leading to the calculation of an overall score indicative of SEWB across the four subscales [21], or whether separate measures are more appropriate. Currently, no studies have employed Rasch analysis to examine the Strong Souls’ scores, representing an opportunity to clarify how the measure functions against the measurement assumptions outlined prior.

This study aimed to examine the scores of the Strong Souls measure against the Rasch measurement model’s assumptions [26] to assess the score validity for use with Aboriginal youth outside of the NT in Australia. As a further indicator of psychometric robustness, construct validity was also assessed by examining correlations between Strong Souls and a 6-item version of the Connor-Davidson Resilience Scale 10 (CD-RISC-10) [27,28,29], which has been recently validated for an Aboriginal youth sample. As an instrument purporting to measure resilience, we expected to find a positive correlation between the CD-RISC and the Strong Souls Resilience factor and a negative correlation with the three Distress-related factors. This instrument was selected as it has shown preliminary evidence towards a valid measure of resilience for use with our sample of Aboriginal youth [29]. Our research questions were:

1. Do scores from the Strong Souls measure meet the Rasch modelling assumptions when used in our sample of Aboriginal youth in WA and NSW, thus providing evidence for reliability and validity in this sample?
2. Is there evidence for concurrent validity between the Strong Souls and the modified CD-RISC?

Evaluating the evidence for these research questions will expand the evidence base available regarding the use of this measure outside of its original validation sample, and contribute to the understanding of SEWB and how it is measured in Aboriginal youth.

## 2. Method

### 2.1. Design

Data were collected cross-sectionally as part of a study investigating resilience and SEWB in Aboriginal youth.

### 2.2. Participants

One hundred and fifty-four participants completed Strong Souls (89 females [58%] and 65 males [42%]). As per Linacre’s [30] guidelines for adequate sample size for Rasch modelling, this sample would expect to find item calibration and person measure estimates stable within ½ logit of accuracy (99% CI). Participants’ ages ranged from 15–25 years. All participants self-identified as Aboriginal and/or Torres Strait Islander and were recruited from two specific study locations. Sixty-three participants (41%) were located rurally on Kamilaroi country (Tamworth/Coledale area, NSW), while the remaining 91 participants (59%) were located on the urban location of Whadjuk Noongar country (Boorloo—Perth metropolitan area, WA). These two study locations were selected as geographically distanced and distinct from the original NT study site, with neither site having had published use or evaluation of Strong Souls, to the authors’ knowledge. The generalisability of our results remains limited to Aboriginal youth in the study locations.

### 2.3. Instruments

#### 2.3.1. Strong Souls Inventory 

The Strong Souls measures SEWB with four distinct factors: anxiety, depression, suicide risk, and resilience. For the anxiety, depression and suicidality subscales, participants indicated how often each item had occurred to them in the past few months using a 4-point Likert scale, with varying point labels depending on the item (e.g., *not much* [1] to *lots of times* [4]; *never* [1] to *lots* [4], etc.). Similarly, for the Resilience subscale, participants indicated how much an item was ‘like’ them on a scale of 1 to 4, whose anchors again differed from item to item (e.g., *always* [1] to *never* [4]; *lots* [1] to *not much* [4]). Item content included “you know someone who is a good person”, “felt so sad nothing could cheer you up?” and “felt so worried it was hard to breathe?”. Alpha reliability coefficients were used to assess reliability when the scale was first developed and piloted [21], with both the overall scale and the four individual factors reporting good internal consistency: overall scale (0.70); anxiety (0.80); depression (0.71); suicide risk (0.73); resilience (0.71).

#### 2.3.2. Connor-Davidson Resilience Scale (CD-RISC)

The CD-RISC is a widely used self-report scale used to measure resilience, validated for use in many different populations. A 6-item modification of the 10-item version has shown preliminary evidence towards acceptability for the current sample [29]. Responses were indicated on a 5-point Likert scale, with higher scores indicating greater resilience. In line with previous research practices [27,28], total scale scores were created by summing ratings of individual items, therefore ranging from 0–24.

### 2.4. Procedure

Ethics approval was granted from the Human Research Ethics Committee of Murdoch University (project no. 2017/122) and the Western Australian Aboriginal Health Ethics Committee (project no. 796). All participants gave informed consent for their participation and were able to stop participation at any point. Two methods were used to collect data. On Whadjuk country, a small number of data points (*n* = 23) were collected online using Qualtrics, a quantitative surveying site [31]. All these participants were over the age of 18 years. The remaining participants on Whadjuk country (*n* = 67) and all participants on Kamilaroi country completed Strong Souls and CD-RISC in-person in the company of a research team member. Due to the potentially distressing content of several Strong Souls items, plus additional items from the wider study that covered topics such as family violence and drugs, the choice was made to have participants under 18 years complete the survey with supervision. This ensured that young participants’ wellbeing and safety could be monitored, and support provided if needed or requested. While over-18s had the option of completing the survey in this manner, they also had the option of completing it individually (online), as it was decided that they would be able to access the resources and relevant local mental health support services linked throughout the survey should they need to. All participants received an AUD 20 gift voucher to compensate them for their time.

### 2.5. Data Analysis

As scores from the Strong Souls were conceptualised to reflect the underlying construct of SEWB, Rasch modelling was used to test this notion of a univariate construct (reflecting SEWB) combining the four outlined subscales. Analyses were conducted using Winsteps software (Version 4.3.4; John M. Linacre, Portland, OR, USA) [32]. Winsteps has traditionally used the Joint Maximum Likelihood Estimation (JMLE) method. JMLE has received criticism from some as a potentially biased or inconsistent method of estimation of item parameters [33]. As such, an updated version of Winsteps (Version 5.1.0; John M. Linacre, Portland, OR, USA) [34], which included the option of using Conditional Maximum Likelihood Estimation (CMLE), was also used to check the impact of the estimation method. As the items of the measure employed varying response category anchors (i.e., the response options on each Likert-style scale were not homogenous across the items, instead varying throughout), a partial-credit polytomous Rasch model was used as the basis for analysis.

To examine the tenability of the Strong Souls’ scoring patterns against the assumptions of the polytomous Rasch measurement model, several specific tests were performed. The criteria we used to evaluate consistency with the assumptions of the Rasch measurement model are outlined in the following subsections.

#### 2.5.1. Dimensionality

To confirm that all scale items were aligned in the same direction on the SEWB construct, measure scores were examined for consistency in their correlation coefficient direction (i.e., positive or negative). Principal component analysis (PCA) was used on the score residuals of the Rasch model to determine whether a univariate structure was apparent. Off-factor item-clusters with an eigenvalue > 2.0, and accounting for more than 10% of the variance in data, were potentially indicative of a multivariate structure [26]. Additionally, disattenuated Pearson correlations between residual clusters were examined, with correlations > 0.71 indicating that the clusters shared more than half their variance and were therefore likely to be measuring the same factor [35].

#### 2.5.2. Response Category Adequacy

Response categories and thresholds were required to advance monotonically (i.e., selecting higher Likert-style scale responses to an item should represent a consistent increase or decrease in underlying SEWB being measured). Additionally, response categories required 10 observations each for adequate functioning [36].

#### 2.5.3. Item Independence

Local item independence was examined using Yen’s Q3* statistic [37] to ensure all item scores were performing independently of one another once the Rasch construct had been accounted for via low inter-item correlations. Yen’s Q3* statistic reflects the difference between mean residual correlations and the largest residual correlation between two items. Smaller correlations (*r* < 0.20) were indicative of item independence [38]. Dependency between items can contribute to creating false inflation of the reliability and therefore overestimate the precision of the measure.

#### 2.5.4. Person and Item Reliability and Separation

The person separation index (PSI) estimated how well a measure can separate participants along a continuum of SEWB levels. Higher PSI indicates that the measure can better distinguish between higher, average, and lower levels of a construct (Acceptable PSI > 2, person reliability index (PRI) > 0.8). The item separation index (ISI) verifies the consistency in difficulty ordering for items in the inventory (e.g., is the item consistently regarded by participants as indicating a higher level of SEWB?). Higher ISI indicates the person sample is large enough to confirm the ordering of items (ISI > 3, and item reliability index (IRI) > 0.9) [39].

#### 2.5.5. Item Fit

The information-weighted fit statistic (Infit) and outlier-sensitive fit statistic (Outfit) mean-square coefficients were examined to assess item misfit. Items were considered acceptable with mean-square coefficients between 0.70 < X < 1.30, and standardised Z coefficients between −2.0 < Z < 2.0 [26]. Item partial correlations were also examined, with r > 0.40 deemed acceptable [26].

#### 2.5.6. Differential Item Functioning (DIF)

DIF was investigated to ensure items were functioning the same across sub-groups (e.g., males and females) in the sample regarding participants’ response patterns. The DIF contrast represented a change in the item measure estimate, based on an external factor (e.g., gender), rather than a difference in the intended construct, potentially distorting interpretation of the measure’s scores as a consequence. The external factors we examined were gender (male/female), location (urban/rural) and age (school-age (15–17) and post-school-age (18–25)). Where Mantel *χ^2^* coefficient contrasts between groups *χ^2^*| > 0.43, and statistically significant (*p* < 0.05), the difference between groups was considered potentially indicative of bias [40].

#### 2.5.7. Concurrent Validity

Provided the assumptions of the Rasch model were met for the Strong Souls’ scale scores, concurrent validity was examined via correlations between Strong Souls and the 6-item CD-RISC. Spearman’s Rho was selected as the statistical method to use for these correlations due to the non-parametric, ordinal nature of the data.

#### 2.5.8. Internal Consistency Estimation

McDonald’s hierarchical omega (ω_h_) was used as a supplementary measure of internal consistency between items. This coefficient was chosen over Cronbach’s alpha (α) based on well-documented biases that exist when using α as an internal consistency measure. Importantly, estimate inflation and attenuation is less likely using ω [41].

## 3. Results

### 3.1. Full-Scale Analyses

#### 3.1.1. Polarity

After reverse-scoring the Resilience subscale items, all items had positive correlations and were therefore aligned in the same direction on the latent variable of SEWB.

#### 3.1.2. Person and Item Reliability and Separation

Upon first inspection of the 25-item scale, person and item reliability and separation indices estimated from the measure’s scores were all acceptable (PSI = 2.21; PRI = 0.82; ISI = 3.42; IRI = 0.92). A negative mean measure score of −1.35 (*SE* = 0.33) indicated participants were less likely to be choosing high scoring responses (i.e., more likely to respond in a manner indicative of higher SEWB).

#### 3.1.3. Dimensionality

**Figure 1:**
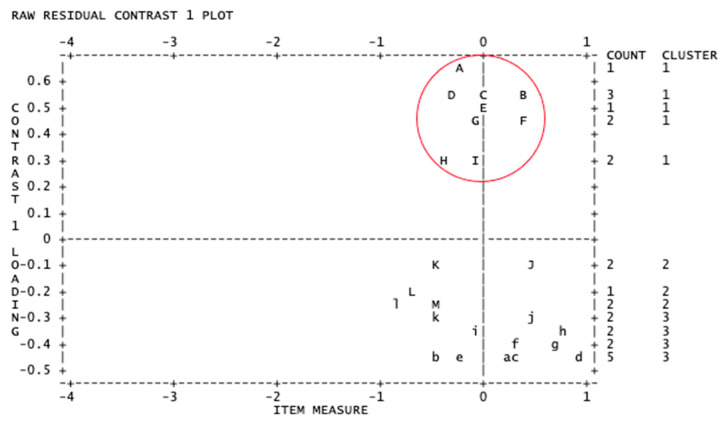
Principal components plot of item loadings. Letters represent items, with cluster A–I representing items 17–25 (Resilience subscale). As demonstrated, the first cluster of the Resilience subscale items has a notably disparate *Y*-axis difference from the remainder of the items that indicate Psychological Distress, evidencing a multidimensional structure to the measure, thereby indicating a unidimensional scale score is likely to be inappropriate.

Principal components analysis of the Rasch model residuals found that the scale scores explained 29.8% of the variance in the participants’ responses. The first off-factor cluster had an eigenvalue of 4.08, above the recommended value of 2.00 [35]. This indicated that the scale scores potentially did not meet the requirement of unidimensionality. Upon examination of the first item cluster, it was clear that these items were the nine items that comprised the Resilience subscale (see Figure 1). The clear vertical separation of the residual clusters demonstrated in Figure 1 was evidence for multidimensionality. Consequently, examination of the measure’s scores’ suitability against the assumptions of the Rasch measurement model continued for two separate scales—Resilience and Psychological Distress (comprising the remaining subscales of Anxiety, Depression and Suicidality).

### 3.2. Psychological Distress (Depression-Anxiety-Suicidality) Subscale

#### 3.2.1. Response Categories

**Figure 2:**
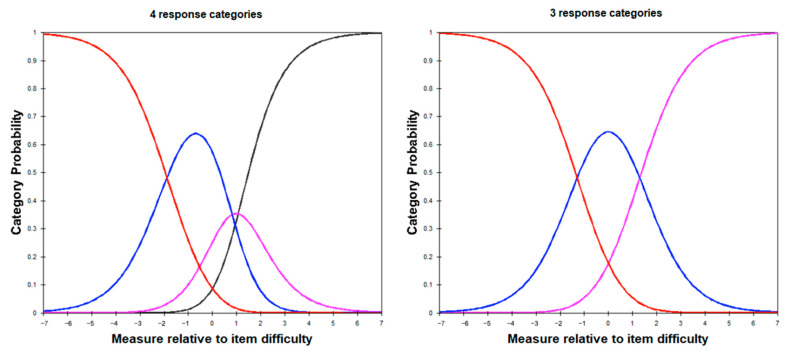
Response category curves for the original response category approach (**left**) and the modified approach with categories 3 and 4 collapsed to form a 3-point scale (**right**). As a partial-credit model was used due to the varying response categories between items, this figure used item 12 as an example.

Using the original response categories of 1–4 (the anchors of which differed across items—*not much/not really/never* and *lots/lots of times*—hence the partial-credit model) resulted in response categories of all items, except item 3, demonstrating inadequate functioning, including less than 10 observations in many higher-distress categories, and excessive noise in the data demonstrated by outfit mean-square values > 2.0 [36]. Disordered thresholds between categories 3 and 4 (*fair bit* and *lots/lots of times*) were also noted in items 2, 5, 6, 12, 13, 14 and 15 (see Figure 2 for example, item 12). This finding indicated that participants were potentially finding it difficult to discriminate between the two categories, and they were therefore not being used as expected. To remedy this, categories 3 (*fair bit*) and 4 (*lots/lots of times*) were collapsed for each of the items (including item 3, for scale consistency), as per the guidelines of Linacre [36]. The improvement in the non-redundancy of the categories is illustrated in Figure 2. Using the 3-point response scale (new category 3: *fair bit/lots*) resulted in improved person and item reliability and targeting (see Table 3).

#### 3.2.2. Dimensionality

The Distress subscales’ dimensionality revealed the raw variance explained by the 16-item scale was 43.1% (eigenvalue = 12.12). An eigenvalue > 2.0 was detected for the first off-factor contrast (2.20; 7.8% of the unexplained variance), which indicated potential multidimensionality. This off-factor cluster of items consisted of the three suicide-related items. However, the disattenuated correlations between this cluster and the remaining items shared a very high variance (i.e., *r* = 1.00). As this suicide-related off-factor cluster comprised a relevant component of distress and the three most difficult to endorse highly items, removing this cluster would have limited the range of the distress measured by the scale’s items. Therefore, these items were retained.

#### 3.2.3. Item Independence

Four items iteratively presented with evidence of local item dependence concerns, thereby violating a key assumption of the Rasch measurement model [42]. In the initial examination, dependence was observed between items 14 (“Have you wished you were dead?”) and 16 (“Have you felt like killing yourself?”: Q3* = 0.56). The substantial similarity between the items’ content gave plausibility to their dependency. Item fit statistics were consulted to determine the item with the poorer item fit characteristics to remove from the scale (see Table 1 fit statistics for dependent items, presented in iterative pairs as each poorer-fitting item was removed). Item 14 had a poorer fit than item 16, and was therefore removed from the final scale. This process occurred four additional times, with Q3* residual correlations > 0.30 between items 8 (“Have you felt so worried it was hard to breathe?”) and 11 (“Have you felt so worried you got dizzy?”; Q3* = 0.44), items 15 (“Felt like hurting yourself?”) and 16 (“Have you felt like killing yourself?”; Q3* = 0.44), and items 2 (“Get angry or wild real quick?”) and 4 (“Had too many bad moods?”; Q3* = 0.39). The item content of each dependent pairing seemed to be conceptually similar for dependence to occur logically. Therefore, the poorer fitting items were removed from the scale (11, 15, 2). Removing these items left the scale with 12 items. Evidence for unidimensionality remained discernible, with an eigenvalue < 2.0 in the first off-factor contrast (1.76; 8.4% of the unexplained variance).

#### 3.2.4. Differential Item Functioning

DIF response bias was evident for the participant categories of gender and location, but not age. For gender, three items had significant DIF contrast coefficients (>0.43 logits, *p* < 0.05): item 6 (“felt so sad nothing could cheer you up”)*,* item 8 (“felt so worried it was hard to breathe”), and item 9 (“felt so worried you got really sweaty”). Females found item 6 significantly more difficult to rate highly in comparison to males (DIF contrast = 0.92, *p* = 0.01). For both items 8 (contrast = −1.03, *p* = 0.005) and 9 (contrast = −0.63, *p* = 0.03), males found it significantly harder to endorse ratings reflecting higher levels of distress compared with females.

For location, two items demonstrated significant DIF (>0.43, *p* < 0.05). Rural participants found item 3 (“hard to focus, thinking all over the place”; 0.66, *p* = 0.01) significantly more difficult to rate highly compared to urban participants. On the other hand, urban participants found item 12 (“got angry or wild and stayed that way for a long time”; −0.64, *p* = 0.03) more difficult to rate.

Removing the item with the highest DIF first found (item 8) resulted in a substantial decrease in the scale person reliability and separation values. Therefore, we determined that removing this item, or any further items with DIF, would be too punitive on the scale’s reliability. Consequently, we retained the items that potentially indicated DIF concerns when checking item fit as follows and note this limitation in the later Discussion.

#### 3.2.5. Item Fit

Item fit statistics are provided in Table 2, with all 12 items meeting the assumptions of the Rasch measurement model. As no consequential differences were found between item fit estimates using JMLE or CMLE methods (see Supplementary Materials—Table S1), JMLE was retained for these item parameters in Table 2.

#### 3.2.6. Person and Item Reliability and Separation

**Figure 3:**
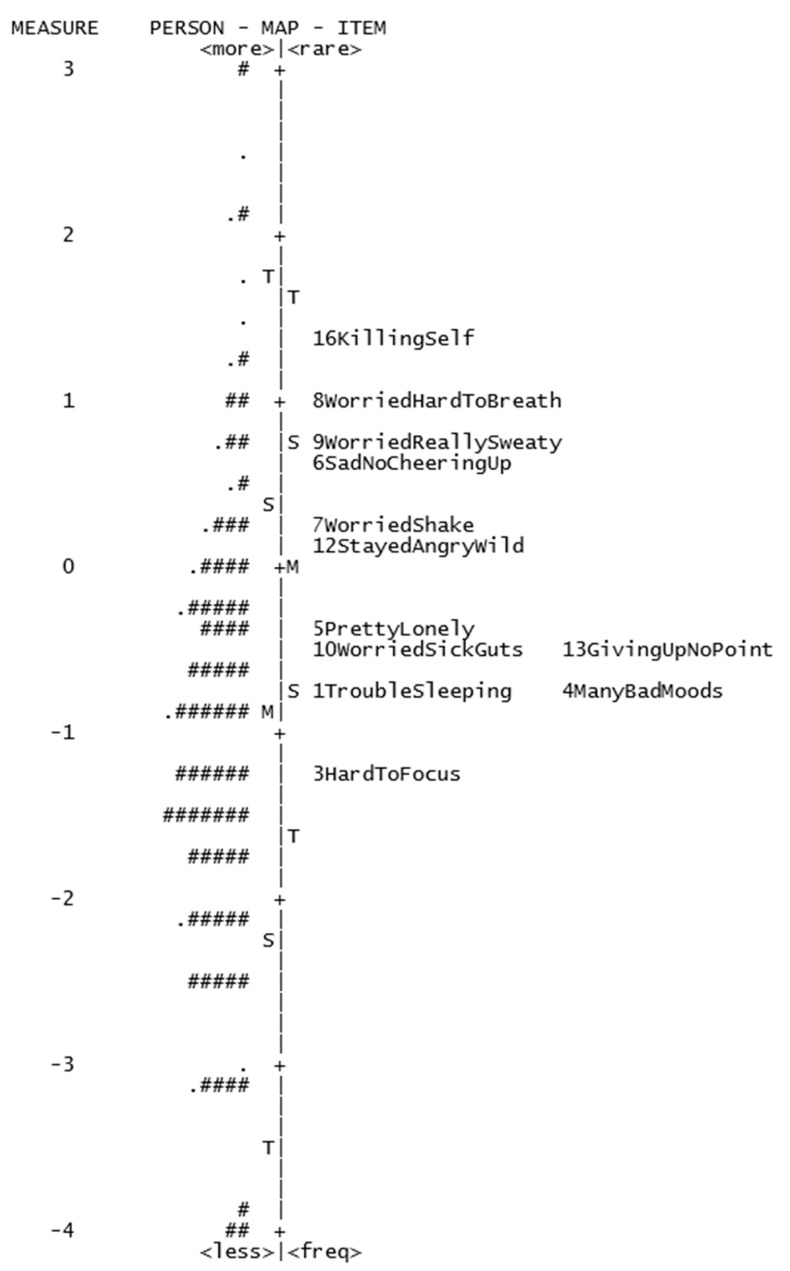
Person-item map of Distress subscale. Each ‘#’ represents 2 participants; each ‘.’ represents 1 participant. *M* = mean, *S* = 1 standard deviation (*SD*), *T* = 2 *SD*s.

The item separation and reliability indexes were well above the recommended thresholds, indicating the ordering of the items’ scores were reliable. Person separation and reliability indexes were slightly below the recommended thresholds (PSI >2; PRI > 0.8; see Table 3). With less than 2 strata of reliability, this suggested that the scale was moving towards order and distinguished between lower/higher Distress scores reliably, but was less reliable in distinguishing any further, i.e., between higher/average/lower scores. As a measure of internal consistency, the ω_h_ value of 0.64 also indicated less-than-adequate scale reliability, based on the person separation and reliability indexes, and the lower ω_h_ value, the Distress subscale remained less reliable in distinguishing between participants (as illustrated in Figure 3).

### 3.3. Resilience Subscale

#### 3.3.1. Response Categories

Under the default response coding system of Strong Souls, a lower total score on the Resilience subscale equated to a higher level of resilience. For clarity and consistency with the Distress subscales’ scoring direction, the coding of the Resilience subscale was reversed to reflect a higher score corresponding to a higher level of the construct.

The response category functioning was not adequate per the guidelines of Linacre [36]. As with the Distress subscale, several items had outfit mean-square values > 2.0, less than 10 observations in lower-Resilience categories, and the middle two thresholds (response categories 2 and 3—*sometimes/little bit/not many* and *most times/fair bit/fair few*) appeared to be generally disordered thresholds. Collapsing categories to remedy this was considered. However, we decided that it did not make semantic sense to collapse categories for this subscale given their anchor wording (e.g., *not really* and *sometimes*) convey varying concepts; therefore collapsing these categories together would not provide an accurate measure. Therefore, the analysis proceeded with the knowledge that response categories were not being used as intended, which was a limitation against the Rasch measurement model assumptions.

#### 3.3.2. Dimensionality

Principal components analysis of the Rasch model residuals found the scale explained 33.2% of the variance. The first off-factor cluster had an eigenvalue of 1.5, indicating the cluster contained less than two off-construct items. This cluster explained 11.3% of the variance of the data. Disattenuated correlations between this first cluster and the remaining cluster were examined, excluding extreme scores (i.e., scores of either 0 or the maximum), with high shared variance (*r* = 0.82) found. These results indicated a univariate structure for the Resilience subscale.

#### 3.3.3. Item Independence

No evidence for local item dependence was found, as all standardised residual correlations between items were marginal (Q3* < 0.30).

#### 3.3.4. Differential Item Functioning

DIF response bias was evident in four items over all three of the investigated participant categories. For age, older participants found it significantly more difficult to highly rate item 24 (“you got lots of friends”) than younger participants (contrast = −0.52 logits, *p* = 0.02). For gender, female participants found item 21 (“you are really into something”) significantly more difficult than male participants to endorse (contrast = 1.02, *p* = 0.002). In contrast, males found item 25 (“When you’re upset you can usually talk to someone about it”) significantly more difficult to endorse than females (contrast = −0.55, *p* = 0.02). For location, item 23 (“you got an older person looking out for you”) was endorsed more easily by rural participants compared with urban-based participants (contrast = −0.59, *p* = 0.03). These significant differences between participant groups’ responses on the four items infringed the Rasch assumption that stipulates that these demographic categories should not impact the participant ordering or participant response to items, as the responses should only be a function of the construct intended to be measured [26].

Removing the items that demonstrated DIF was considered; however, when tested, it resulted in a considerable decrease in person and item targeting and reliability—well-below values acceptable to the Rasch model. Hence, these items were retained in the final Resilience scale, acknowledging that the items were not functioning as equal measures of the construct across different participant categories.

#### 3.3.5. Item Fit

JMLE was used to assess item fit, although a comparison of item fit estimates using JMLE and CMLE methods highlighted no meaningful differences (see Table S2). The infit and outfit coefficients of one item (18; “you know about white fella ways”) suggested potential misfit (i.e., mean-square estimate outside of 0.70 < X < 1.30; [26]). Even though removing this item from the measure worsened overall PRI/PSI, we decided the evidence for misfit still warranted its removal. This action was supported by previous findings documented by Thurber et al. [20], where item 18 was also removed from latter versions of the Resilience scale based on face validity and reliability concerns. Accordingly, we have used an 8-item measure for the forthcoming analyses. Item fit statistics with item 18 removed are provided in Table 4.

#### 3.3.6. Person and Item Reliability and Separation

The Resilience subscale did not meet the Rasch assumptions for item and person separation and reliability. Person reliability (0.59) and separation (1.20) coefficients suggested that the items in the 9-item Resilience subscale did not demonstrate adequate person reliability. This finding was also reflected in a low hierarchical omega (ω_h_) reliability coefficient of 0.61. Item reliability (0.81) and separation (2.05) coefficients indicated potential concerns regarding item ordering reliability. Poor scale targeting was reflected in an estimated person-measure value of 1.37 (SE = 0.60), suggesting participants found items generally easier to endorse.

### 3.4. Concurrent Validity

Only concurrent validity between the Distress subscale and the CD-RISC was assessed. This decision was made based on the lack of evidence from the Rasch modelling procedure outlined prior, demonstrating the Resilience subscale as a well-functioning measure of resilience in our sample. As the 12-item Distress subscale showed adequate ISI and IRI, and PSI/PRI approaching adequate values, a Spearman’s rank correlation was performed to assess the strength of the relationship between the Distress subscale and the modified version of the CD-RISC. We expected to find a negative relationship between the Distress subscale and the CD-RISC. Results of the Spearman’s correlation indicated that there was a significant negative association between CD-RISC scores and Distress scores, r_s_(145) = −0.34, 95% BCa CI[−0.491, −0.144], *p* < 0.001. This r value illustrated a small effect size [43].

## 4. Discussion

This study provides novel evidence regarding the functioning of the Strong Souls instrument beyond its original sample in the Northern Territory [21]. Our results indicate that for our sample in WA and NSW, the scale does not function as one unidimensional measure of SEWB. When analysing Resilience and Psychological Distress as two separate scales, we found that both measures had difficulty meeting the Rasch measurement model’s assumptions, indicating less-than-adequate psychometric properties against these model assumptions. However, these findings still provide practical options for moving forward to improve the utility of the scale and raise important and timely questions regarding how different wellbeing-related constructs are conceptualised and the implications this has on measurement, theory, and practice.

### 4.1. Resilience Subscale

As a stand-alone measure, the Resilience subscale did not meet Rasch measurement expectations for the person and item separation reliabilities. These values were also echoed by the CTT perspective with a low hierarchical omega coefficient (*ω_h_*,), demonstrating poor saturation of the latent construct by the items. Along with several items functioning differently across participant groups, and a response category system that was not working sufficiently, the Resilience subscale requires further adjustments to items and response categories before it can be used confidently as a reliable measure for the current sample.

### 4.2. Psychological Distress Subscale

On the other hand, the Distress subscale, while also requiring modifications before it can be considered a robust measure for this sample, illustrated more mixed results in terms of its adherence to Rasch specifications. Notably, the item separation and reliability coefficients were well above the recommended Rasch minimum values, indicating that the scale had a broad enough range of items and adequate sample size to confirm the item difficulty hierarchy, implying good construct validity. However, there remained several aspects of the scale that were lacking. The scale items did not target the sample’s distress levels as expected by the Rasch model. This is illustrated in Figure 3 and is reflected in the low person separation and reliability values, highlighting the lack of sensitivity of the scale to distinguish between high and low distress between participants. Additionally, with five items displaying DIF, significant differences between responses hinged on participant group categories of gender, location, and age (rather than distress level) and point to a scale that is not measuring the intended construct without the influence of spurious factors.

### 4.3. Theoretical and Practical Implications

The Strong Souls scale’s multidimensionality underscores the importance of clearly defining constructs, such that items can accurately measure the intended construct. This highlights the necessity for a clear theoretical basis with well-defined, operationalised constructs in the process of psychometric scale development [44]. The original scale authors sought to create a scale that measured SEWB, providing a broad depiction of health from a specifically Aboriginal perspective, providing insights rooted in holistic conceptualisations of health and wellbeing [21]. This involved a view of mental health linked to connection to Country, kinship, and physical, cultural, emotional and spiritual wellbeing. However, linking the subscale factors and items to a specifically articulated and measurable definition of SEWB was less discernible in this original work. Recent explorations of SEWB have further expanded on the construct as a fully articulated model. For example, the seven domains of SEWB outlined by Gee et al. [12] clearly outline the multifaceted concept, also incorporating the necessity of social and historical determinants in the conceptualisation of SEWB. The constructs of depression, anxiety, and suicide risk, while concepts relevant to the overall wellbeing discourse, are not explicitly articulated with respect to the construct of SEWB. Clearly outlined in Gee et al.’s [12] model, and in relevant policy documents such as the *National Strategic Framework for Aboriginal and Torres Strait Islander Peoples’ Mental Health and Social and Emotional Wellbeing* is a strong focus on the strengths, endurance, and cultural and spiritual connections of Aboriginal peoples as core to the SEWB perspective [15]. This strengths emphasis was somewhat obscured with the Distress constructs’ inclusion in the Strong Souls, which appears to frame them as key concepts of SEWB. It is perhaps inexact, without qualifiers, to align the Distress constructs of anxiety, depression and suicide risk to an instrument characterised to measure SEWB specifically. While not dismissing the role and importance of addressing the need to identify and reduce symptoms associated with mental health disorders, equating the absence of these symptoms to the holistic connectedness with culture, family, community, spirituality and Country that embodies the SEWB construct may not be the clearest way to assess the construct. This misalignment with a strengths-focussed SEWB construct may explain the lack of unidimensionality of the scale, with the Distress factors not aligning with the overarching construct of SEWB.

The nature of the relationship between the construct of resilience and SEWB overall also remains ambiguous, impacting the instrument’s robustness and validity. How the Strong Souls conceptualises resilience, and how this construct is proposed to interact with SEWB was not clearly defined in the reporting of the scales’ construction. This is an integral step for a rigorous scale development process. Researchers have argued that resilience is not necessarily the absence of distress or poor mental health symptoms, which may also help to explain the lack of unidimensionality between Resilience and Distress subscales—one is not necessarily the inverse of the other, or on the same continuum [45,46]. A clear, operationalised definition of resilience would help clarify this. Widely accepted definitions of resilience from seminal resilience researchers stipulate the inclusion of adversity, setback or stress as an essential feature in naming and identifying the positive outcome or process of resilience (see [47,48]). This notion is not clear in the Strong Souls’ conceptualisation.

### 4.4. Limitations and Future Directions

Given the poor person reliability and separation statistics, both the Resilience and the Psychological Distress subscales would benefit from the development of additional items, to increase the length and breadth of the instruments. Generating items focussed on tapping into higher levels of resilience and lower levels of distress would potentially ameliorate the targeting issues seen in both scales. Generation of alternate items is also necessary to address the several instances of DIF, ensuring that new items are being interpreted and responded to without unintended factors like gender, age or location impacting responses. However, for a functional, univariate measure of SEWB, closer consideration of this construct as a whole, and the range of items that sufficiently cover the breadth of this construct is required.

While Strong Souls had mixed evidence against the assumptions of the Rasch measurement model, the scale and the current findings provide a solid basis to work further to conceptualise and measure SEWB and related constructs. From our results, these two subscales do not appear to be functioning together as measuring one total construct of SEWB for our sample. Given the nature of the multifaceted construct of SEWB, a valuable approach to measuring it may involve several separate scales addressing different aspects of the construct. However, for a measure with clear construct validity, operationalised definitions of all constructs and factors must be clearly outlined. In particular, consideration of how resilience is conceptualised (and thus measured) would have implications for how the scale and items are presented.

## 5. Conclusions

SEWB is an important construct with relevance and meaning for young Aboriginal people, embodying notions of wellbeing in language and concepts that begin to dismantle and decolonise the Western deficit-based notions of health that dominate the Australian landscape [7,12]. The measurement of SEWB is therefore important in providing accurate and relevant depictions of how things are for Aboriginal youth, illustrating where further support may be required, and what factors within and around young people are helping them thrive. Strong Souls is an instrument that proposes to capture SEWB. For our sample of young Aboriginal people in metropolitan WA and rural NSW, the Strong Souls instrument in its current form does not function adequately as a univariate measure of the SEWB construct, with both the Resilience and Psychological Distress subscales requiring further consideration in item generation and their theoretical underpinnings before it can be used as a robust and reliable instrument in our sample. Further attention is required to quantify and measure SEWB in accurate and relevant ways.

## Figures and Tables

**Table 1:** Fit statistics for dependent items.

Fit Statistics		Items							
		Iteration 1		Iteration 2		Iteration 3		Iteration 4	
		14	16	8	11	15	16	2	4
Infit	MnSq	0.81	0.76	0.97	1.01	1.08	0.82	1.20	0.93
	ZStd	−1.65	−1.62	−0.17	0.10	0.61	−1.13	1.93	−0.70
Outfit	MnSq	0.75	0.59	0.86	1.02	0.99	0.67	1.29	0.97
	ZStd	−1.53	−1.64	−0.76	0.16	0.01	−1.24	2.39	−0.25

Note. Infit = overfit coefficient; Outfit = underfit coefficient; MnSq = mean-square coefficient; ZStd = *Z* score. Each “iteration” depicts fit statistics as each item was removed due to item dependency. Numbers in bold were the items removed in the iterative process.

**Table 2:** Final 12 Distress item difficulties, fit, and measure correlations in order of descending misfit.

Item	Measure	Infit		Outfit		*r*_partial_
		MnSq	ZStd	MnSq	ZStd	
9—Felt so worried you got sweaty	0.78	1.24	1.86	1.11	0.69	0.50
1—Trouble sleeping?	−0.75	1.11	1.02	1.13	1.15	0.58
4—Too many bad moods?	−0.76	1.05	0.47	1.09	0.80	0.60
8—So worried it was hard to breathe?	1.01	1.08	0.69	0.96	−0.15	0.55
3—Trouble focusing, thoughts all over the place?	−1.30	1.03	0.29	1.01	0.14	0.60
5—Lonely most of the time?	−0.39	0.97	−0.29	1.03	0.25	0.64
10—So worried you felt sick in the guts?	−0.53	1.03	0.27	1.03	0.32	0.60
13—Felt like giving up?	−0.48	1.00	0	0.97	−0.28	0.62
7—Felt so worried you shake?	0.26	0.95	−0.45	0.97	−0.17	0.62
12—Get angry/wild and stay like that for ages?	0.10	0.94	−0.56	0.96	−0.28	0.63
16—Felt like killing yourself?	1.43	0.86	−0.83	0.87	−0.36	0.57
6—Felt so sad and nothing could cheer you up?	0.62	0.79	−2.12	0.75	−2.14	0.68

Note. Measure = item difficulty relative to underlying factor; Infit = overfit coefficient; Outfit = underfit coefficient; MnSq = mean-square estimate; ZStd = *Z* score; *r*_partial_ = partial correlation coefficient between item score and remaining item scores. Item wording from Thomas et al. [21].

**Table 3:** Person and item measures, separation, and reliability indexes for six iterations of the Distress subscale of Strong Souls.

Scale Modification	Measure (SE)	PSI	PRI	ISI	IRI
16 items—Original coding	−1.70 (0.45)	2.12	0.82	4.72	0.96
16 items—Revised 1233 coding	−0.99 (0.49)	2.23	0.83	5.01	0.96
15 items—Item 14 removed	−0.94 (0.50)	2.14	0.82	5.06	0.96
14 items—Items 14 and 11 removed	−0.94 (0.51)	1.98	0.80	5.18	0.96
13 items—Items 14, 11, and 15 removed	−0.84 (0.52)	1.94	0.79	4.88	0.96
12 items—Items 14, 11, 15, and 2 removed	−0.90 (0.56)	1.90	0.78	4.93	0.96

Note. Measure = difficulty coefficient, SE = standard error, PSI = person separation index, PRI = person reliability index, ISI = item separation index, IRI = item reliability index.

**Table 4:** Resilience item difficulties, fit, and measure correlations in order of descending misfit.

Item	Measure	Infit		Outfit		*r*_partial_
		MnSq	*Z*Std	MnSq	*Z*Std	
20—Laugh and makes jokes?	−0.09	1.16	1.22	1.25	1.61	0.48
25—You’ve got someone to talk to when you’re upset.	0.40	1.12	1.03	1.09	0.75	0.60
21—You’re into something (like music, fishing, football, etc.)	−0.47	1.03	0.23	0.90	−0.45	0.50
19—You know someone who’s a good person.	0	1.00	0.01	0.99	−0.03	0.50
17—Your family is strong, and they help each other.	−0.41	0.98	−0.13	0.94	−0.38	0.56
22—You’re a good son/daughter to your family.*	0.03	0.95	−0.35	0.93	−0.55	0.60
24—Lots of friends?	0.39	0.93	−0.53	0.95	−0.42	0.65
23—An older person is looking out for you.	0.16	0.84	−1.15	0.70	−1.29	0.58

Note. Measure = item difficulty relative to underlying factor; Infit = overfit coefficient; Outfit = underfit coefficient; MnSq = mean-square estimate; *Z*Std = *Z* score; *r*_partial_ = partial correlation coefficient between item score and remaining item scores. Item wording from Thomas et al., 2010. *Item wording slightly altered for WA cohort based on cultural advice from local Aboriginal consultative group. Meaning remained the same.

## Data Availability

The data presented in this study is not publicly available due to ethics restrictions. Qualified and interested readers can contact the Murdoch University Human Research Ethics Committee for further information.

## Supplementary Materials

The following are available online at https://www.mdpi.com/article/10.3390/ijerph18168425/s1, Table S1: Comparing two estimation methods: Final 12 Distress item difficulties, fit, and measure correlations in order of descending misfit, Table S2: Comparing two estimation methods: Final 8 Resilience item difficulties, fit, and measure correlations in order of descending misfit.
