# Supplementary material for: Measuring Social and Emotional Wellbeing in Aboriginal Youth Using Strong Souls: A Rasch Measurement Approach
Source: Int J Environ Res Public Health. 2021 Aug 10;18(16):8425. doi: 10.3390/ijerph18168425 (PMC8391716; doi:10.3390/ijerph18168425)
Supplement: Supplementary file 1 [file ijerph-18-08425-s001.zip › ijerph-1293317-supplementary.pdf]

Table S1. Comparing two estimation methods: Final 12 Distress item difficulties, fit, and measure correlations in order of descending misfit.

Item	JMLE Measure	CMLE Measure	JMLE				CMLE				$r_{partial}$
			Infit		Outfit		Infit		Outfit		
			MnSq	ZStd	MnSq	ZStd	MnSq	ZStd	MnSq	ZStd	
9 – Felt so worried you got sweaty	0.78	0.71	1.24	1.86	1.11	0.69	1.24	1.87	1.12	0.72	0.50
1 – Trouble sleeping?	−0.75	−0.68	1.11	1.02	1.13	1.15	1.12	1.12	1.15	1.29	0.58
4 – Too many bad moods?	−0.76	−0.70	1.05	0.47	1.09	0.80	1.05	0.54	1.10	0.90	0.60
8 – So worried it was hard to breath?	1.01	0.92	1.08	0.69	0.96	−0.15	1.08	0.69	0.96	−0.16	0.55
3 – Trouble focusing, thoughts all over the place	−1.30	−1.18	1.03	0.29	1.01	0.14	1.02	0.24	1.01	0.12	0.60
5 – Lonely most of the time?	−0.39	−0.37	0.97	−0.29	1.03	0.25	0.98	−0.18	1.05	0.42	0.64
10 – So worried you felt sick in the guts?	−0.53	−0.48	1.03	0.27	1.03	0.32	1.02	0.23	1.03	0.30	0.60
13 – Felt like giving up?	−0.48	−0.43	1.00	0	0.97	−0.28	0.99	−0.2	0.97	−0.29	0.62
7 – Felt so worried you shake?	0.26	0.24	0.95	−0.45	0.97	−0.17	0.95	−0.44	0.98	−0.14	0.62
12 – Get angry/wild and stay like that for ages?	0.10	0.10	0.94	−0.56	0.96	−0.28	0.94	−0.58	0.96	−0.30	0.63
16 – Felt like killing yourself?	1.43	1.29	0.86	−0.83	0.87	−0.36	0.86	−0.84	0.86	−0.43	0.57
6 – Felt so sad and nothing could cheer you up?	0.62	0.57	0.79	−2.12	0.75	−2.14	0.78	−2.18	0.75	−2.22	0.68

Note. Measure = item difficulty relative to underlying factor; JMLE = Joint Maximum Likelihood Estimation; CMLE = Conditional Maximum Likelihood Estimation; Infit = overfit coefficient; Outfit = underfit coefficient; MnSq = mean-square estimate; ZStd = *Z* score; $r_{partial}$ = partial correlation coefficient between item score and remaining item scores. Item wording from Thomas et al., 2010.

Table S2. Comparing two estimation methods: Final 8 Resilience item difficulties, fit, and measure correlations in order of descending misfit.

Item	JMLE Measure	CMLE Measure	JMLE				CMLE				$r_{partial}$
			Infit		Outfit		Infit		Outfit		
			MnSq	ZStd	MnSq	ZStd	MnSq	ZStd	MnSq	ZStd	
20 – Laugh and makes jokes?	−0.09	−0.06	1.16	1.22	1.25	1.61	1.16	1.22	1.26	1.83	0.48
25 – You’ve got someone to talk to when you’re upset.	0.40	0.35	1.12	1.03	1.09	0.75	1.14	1.21	1.13	1.13	0.60
21 – You’re into something (like music, fishing, football et.)	−0.47	−0.40	1.03	0.23	0.90	−0.45	1.02	0.21	0.90	−0.46	0.50
19 – You know someone who’s a good person	0	−0.02	1.00	0.01	0.99	−0.03	0.98	−0.14	0.98	−0.10	0.50
17 – Your family is strong, and they help each other.	−0.41	−0.38	0.98	−0.13	0.94	−0.38	0.97	−0.22	0.94	−0.41	0.56
22 – You’re a good son/daughter to your family.	0.03	0.002	0.95	−0.35	0.93	−0.55	0.94	−0.44	0.93	−0.61	0.60
24 – Lots of friends?	0.39	0.31	0.93	−0.53	0.95	−0.42	0.92	−0.67	0.94	−0.48	0.65
23 – An older person is looking out for you.	0.16	0.18	0.84	−1.15	0.70	−1.29	0.89	−0.75	0.77	−1.08	0.58

Note. Measure = item difficulty relative to underlying factor; JMLE = Joint Maximum Likelihood Estimation; CMLE = Conditional Maximum Likelihood Estimation; Infit = overfit coefficient; Outfit = underfit coefficient; MnSq = mean-square estimate; ZStd = *Z* score; $r_{partial}$ = partial correlation coefficient between item score and remaining item scores. Item wording from Thomas et al., 2010.
